# Prevalence of High Blood Pressure, Heart Disease, Thalassemia, Sickle-Cell Anemia, and Iron-Deficiency Anemia among the UAE Adolescent Population

**DOI:** 10.1155/2013/680631

**Published:** 2013-03-27

**Authors:** Caroline Barakat-Haddad

**Affiliations:** University of Toronto Scarborough, 1265 Military Trail, Toronto, ON, Canada M1C 1A4

## Abstract

This study examined the prevalence of high blood pressure, heart disease, and medical diagnoses in relation to blood disorders, among 6,329 adolescent students (age 15 to 18 years) who reside in the United Arab Emirates (UAE). Findings indicated that the overall prevalence of high blood pressure and heart disease was 1.8% and 1.3%, respectively. Overall, the prevalence for thalassemia, sickle-cell anemia, and iron-deficiency anemia was 0.9%, 1.6%, and 5%, respectively. Bivariate analysis revealed statistically significant differences in the prevalence of high blood pressure among the local and expatriate adolescent population in the Emirate of Sharjah. Similarly, statistically significant differences in the prevalence of iron-deficiency anemia were observed among the local and expatriate population in Abu Dhabi city, the western region of Abu Dhabi, and Al-Ain. Multivariate analysis revealed the following significant predictors of high blood pressure: residing in proximity to industry, nonconventional substance abuse, and age when smoking or exposure to smoking began. Ethnicity was a significant predictor of heart disease, thalassemia, sickle-cell anemia, and iron-deficiency anemia. In addition, predictors of thalassemia included gender (female) and participating in physical activity. Participants diagnosed with sickle-cell anemia and iron-deficiency anemia were more likely to experience different physical activities.

## 1. Introduction

Effective response to emerging health issues requires public health programs and policies that evolve based on trends in population health and health risk factors [1]. Health surveillance plays a key role by gathering information and shedding light on spatial and temporal variations in health measures. Health surveillance is particularly relevant in countries that exhibit rapid economic development, given that changes to population health measures and risk factors are imminent and largely dynamic [2]. Thus, in countries that exhibit rapid economic growth, research studies should aim to examine the links between social, demographic, and economic contextual factors and health, in order to guide policy and address new emerging health issues [3]. 

The United Arab Emirates (UAE) is a country that has experienced unprecedented growth in the past forty years. The UAE currently ranks 12th among world economies in relation to gross national income per capita [4]. The federation is situated in the southeast of the Arabian Peninsula in Southwest Asia on the Persian Gulf. It consists of Abu Dhabi (the nation's capital), Dubai, Sharjah, Ajman, Umm al-Quwain (UAQ), Ras al-Khaimah (RAK), and Fujairah. Using oil revenue, the UAE seeked to reduce reliance on oil and to diversify its economy from a conventional, labor-intensive economy to one based on knowledge, technology, and skilled labor. This fast pace economic development caused a large influx of migrant workers to the UAE, making the UAE one of the most ethnically diverse countries in the world. According to census data 2005, only 20.1% of the population (estimated at over 4 million) is of Emirati national origin. The remainder consists of other Arabs and Iranians (23%), South Asians (50%), and others including expatriates from Europe, North America, and East Asia (8%) [5, 6]. 

With economic growth, inevitable influences on lifestyle, the built environment, and opportunities and access to resources took place [7]. For the Emirati population, these changes resulted in social transformations that affected the traditional way of living and increased the prevalence of certain risk factors. Many of these risk factors are known to be associated with the development of chronic diseases [8]. Changes to the socioeconomic environment and lifestyle influenced food consumption (i.e., increase in the consumption of meat, poultry, sugar, and fat). In addition, these changes led to heavy reliance on car transportation, sedentary lifestyles, domestic help, and a preference for indoor activities. Indeed, research findings point to the low levels of physical activity among Emirati postsecondary and school-aged females [9, 10]. UAE-based research findings highlight concerns for diverse health outcomes such as asthma, obesity, diabetes, anemia, motor vehicle injuries, cardiovascular diseases, and other genetic diseases [8, 11–14]. Cardiovascular diseases are a major concern and the leading cause of overall death in the UAE. In 2004, cardiovascular diseases constituted 35.3% of all deaths that occurred in Dubai [15]. Data on causes of death indicate that 27% of death in Abu Dhabi was attributed to cardiovascular diseases in 2010 [16]. Both obesity and diabetes are high risk factors associated with cardiovascular diseases and are more recently affecting the young UAE population [12]. With high blood pressure prevalence in the UAE much higher than the world average at 41% of the population, health professionals from Dubai have called for a national screening for hypertension that targets secondary school children [17]. Medical professionals have attributed the disproportionate number of young heart patients in the UAE to the high prevalence of diabetes and unhealthy lifestyles including smoking, sedentary lifestyle, stress, and unhealthy eating habits [18]. Other social risk factors include increased genetic predisposition due to marriages within families and the flux of migrant workers who may not be aware of certain health risks such as smoking.

Consanguineous marriages are common practice among the national UAE population and contribute to the prevalence of blood disorders such as thalassemia. Thalassemia is a major public health issue in the UAE with one in 12 persons said to be a thalassemia carrier [19]. Earlier findings estimate the prevalence of alpha thalassemia, G6PD deficiency, beta thalassemia, and sickle haemoglobin at 16.5, 8.7, 1.7, and 1.9% among the adult UAE population [20]. More recently, the prevalence of anemia, iron depletion, iron-deficiency anemia, G6PD deficiency, sickle cell trait, and beta thalassemia among Arabic preschool children were estimated at 36.1%, 26%, 9.9%, 9.1%, 4.6%, and 8.7%, respectively [13]. In fact, the prevalence of thalassemia in the national UAE population is one of the highest in the world [21]. 

Although the entire UAE population is affected by these fast-paced transformations and lifestyle changes, the impact of these changes on the UAE adolescent population is of particular relevance for two main reasons. First, evidence increasingly suggests that adolescents may be disproportionately impacted by social and physical environmental changes [22]. Second, the adolescent population constitutes over 16% of the UAE population [6]; thus health effects on this subpopulation will result in a large burden on healthcare. Furthermore, since most UAE-based health research tends to be based on small-scale studies in defined geographical areas within the federation, there is a clear need for a national study that examines the health of the adolescent UAE population. In order to address this need, two researchers in collaboration with the UAE Ministry of Education developed a research program entitled “A national study of population health in the UAE (NSPHUAE) (2007–2009).” The program consisted of developing and administering a comprehensive large-scale cross-sectional survey on over 6,300 adolescent children that attend public and private schools in the seven Emirates of the federation, including 9 educational zones, thereafter referred to by geographical regions.

The developed survey consisted of two main components. The first component collected data on active and smoking behaviors, type and frequency of tobacco use, hygiene practices, health promotion behaviors, fish consumption, ultraviolet exposure, injury including severity and location, physical inactivity, wireless usage frequency as proxy for exposures to electromagnetic fields, frequency and type of physical activity patterns, medication use, self-reported sensory health, school absenteeism, functional capacity, quality of life, self-reported medical diagnoses, symptoms in relation to respiratory health, healthcare access and use, road traffic risk factors, and demographic and socioeconomic data. The second component collected data related to residential mobility and locations, residential characteristics including age of residents and exposures to renovations, pesticides, insecticides, source of water supply, proximity to industries/overhead power lines, concern over air pollution, and medical diagnoses for parents and siblings. Data was analyzed using univariate analysis and Bivariate analyses that examined differences in the prevalence of high blood pressure, heart disease, thalassemia, sickle-cell anemia, and iron-deficiency anemia across the Emirates, gender, and ethnicity. Multivariate modeling examined the relative contribution of explanatory sociodemographic and lifestyle variables in predicting the occurrence of various health outcomes.

This paper is the first of a series of publications that disseminates results of the *NSPHUAE*. The paper addresses three main objectives: (I) to determine the prevalence of medical diagnoses related to blood disorders—any thalassemia, sickle cell anemia, and iron-deficiency anemia-, and blood circulatory conditions—high blood pressure and heart disease—among the UAE adolescent population; (II) to examine spatial differences in the prevalence of these medical diagnoses in relation to the geographical regions; (III) to examine prevalence differences in relation to gender and among the local (Emirati) and expatriate adolescent UAE subpopulations; (IV) to determine significant socioeconomic, demographic, and lifestyle predictors of blood disorders, high blood pressure, and heart disease among the UAE adolescent population. 

## 2. Method

 The study included a randomly selected stratified sample of 147 public and private secondary schools. School enrolment data from the UAE Ministry of Education (2005-2006 for private schools and 2006-2007 for public schools) was used in the stratification strategy. The appropriate boards within the UAE Ministry of Education and the UAE Ministry of Health approved the study prior to survey administration. The researchers held training workshops for social workers, employed by the UAE Ministry of Education. The social workers administered the cross-sectional survey on students from three randomly selected classes from each participating school (one class from each of grades 10, 11, and 12). The first component of the survey was administered on the adolescent participants during a spare period in a classroom setting. Social workers collected the completed survey at the end of the defined one-hour period. The second component of the survey was sent home with the student overnight, in order for the student to seek parental assistance in completing it. When the student participants returned it the following day, a participant identification code allowed it to be matched to the first component. Social workers packaged the completed surveys in preassigned boxes and sent the boxes to the UAE Ministry of Education, where researchers picked them up. Researchers entered and analyzed the data using the Statistical Package for the Social Science (SPSS-v16).

### 2.1. Exposure Variables Used in the Analysis

 In order to address the study objectives, analysis included answers to items that collect data related to sociodemographic, residential, and lifestyle. Sociodemographic variables included sex, birth year, parental marital status, ethnicity, and socioeconomic status. Two variables categorized ethnicity by whether the participant is local (Emirati) or expatriate, and whether the participant falls into one of the following categories: UAE local, GCC (includes participants from GCC (Gulf Cooperation Council) countries or GAFTA (Greater Arab Free Trade Area) including Kuwait, Kingdom of Saudi Arabia, Oman, Qatar, Bahrain, and Yemen), Arab/Middle East (participants from Lebanon, Syria, Jordan, Palestine, and Iraq), Arab/Africa (includes Egypt, Tunisia, Morocco, Algeria, Libya, Sudan, and Somalia), South East Asia (includes India, Pakistan, Bangladesh, Sri Lanka, Philippines, and Indonesia), Western (Europe, USA, Australia, and Canada), no nationality, and others (all other nationalities). Similar cultures, traditions, ancestral linkages, or geographical origins accounted for the categorization of these ethnicities. Data related to annual family income, father's employment status and education level, type of school attended (private versus public), number of individuals that reside in the household, number of bedrooms in the main residence, and property tenure assessed socioeconomic status. Family income was included as a Bivariate variable with those that report an annual family income at or below AED15,000 in one category versus those that report a family income greater than AED15,000. This cut-off was selected based on data from the UAE Ministry of Economy indicating that the average monthly income for Emirati households is AED36,438.10 and that for expatriate households is AED15,074.30 [23]. The ratio for the number of individuals that reside in the household over the number of bedrooms in the main residence assessed residential crowding, which was determined using a ratio greater than 2.

 Eight items collected data related to residential characteristics and exposures. The educational zone of the school that the participant attended categorized the location of residence into one of 9 geographical regions: city of Abu Dhabi (AUH), western region of AUH (thereafter the western region), Al Ain AUH (thereafter Al Ain), Dubai, Sharjah, Ajman, Ras al-Khaimah (RAK), Fujairah, and Umm al-Quwain (UAQ). One item asked whether the property of residence was built prior to 1988; this variable was used as proxy for exposure to lead in residential paint and other construction materials that are known to impact health and that had been used in the UAE in older residences. Items collected data on whether the place of residence is subject to regular use of pesticides or insecticides, pest infestations, and whether they live near to industrial plants, gas stations, dumpsites, or construction or near to overhead power lines or plants.

Items related to one's lifestyle collected data on smoking, exposure to smoking, illegal drug use, unconventional drug use, and physical activity. This included questions on whether they ever smoked cigarettes, tobacco, shisha (waterpipe), or midwakh. They were also asked whether participants smoked cigarettes, tobacco, shisha, or midwakh in the past 30 days. Active smokers consisted of participants who reported occasional or daily smoking of cigarette, tobacco, shisha, or midwakh in the past 30 days. Items collected data on the age when participants first smoked any kind of tobacco, the frequency that they were exposed to tobacco at home or among their friends, and the age when they were regularly exposed at least twice a week to smoking at home or when with friends. Passive smokers consisted of those who reported occasional or daily exposure to smoking. In relation to substance abuse, two items asked whether participants have ever used illegal drugs such as marijuana, hashish, or cocaine and whether they ever purposely smelled gasoline, glue, fumes, correctors, car exhaust, or burning black ants. These “other” forms of substance abuse are common knowledge in the UAE adolescent population. A series of questions that asked participants whether they participated in any of a number of 17 activities in the past 12 months assessed participation in physical activity. 

### 2.2. Outcome Variables

Self-reported responses to items that collected data on medical diagnoses assessed health outcome variables. These included items on whether or not a doctor or health professional had ever told participants that they have any of the following conditions: high blood pressure or hypertension, heart disease, thalassemia, sickle-cell anemia, any other type of anemia, and any other major health diagnosis. An open-ended item provided participants an opportunity to specify their medical diagnosis, in relation to heart disease, anemia, and other medical diagnosis. 

### 2.3. Statistical Analysis

Data were analyzed using SPSS-v16. Univariate analysis consisted of frequency analyses for high blood pressure, heart disease, thalassemia, sickle-cell anemia, and iron-deficiency anemia. Bivariate analyses were used to identify significant differences in the prevalence of these medical conditions across the Emirates, in relation to gender and among locals versus expatriates. The chi-square value and the *F* statistic were used to determine measures of association, with a *P* value less than 0.05 determining statistical significance. 

Conditional logistic regression modeling was used to assess the relative contribution of explanatory sociodemographic, residential, and lifestyle variables in predicting the occurrence of the health outcomes. For each of the modeled health outcomes, forward stepwise entry of groups of variables was conducted; thus, explanatory variables were entered into the model one step at a time in order to identify significant predictors of the health outcome using a significance level of *P* ≤ 0.1. For categorical independent variables, one category was chosen to be the reference category, with each category of the variable then compared to the reference category (e.g., in relation to the relative contribution of family income, participants with family income above AED15,000 are compared to those whose family income at or below AED15,000, the latter being the reference category). This process resulted in a model that included significant explanatory variables (thereafter predictors) that explain the health outcome. The specificity (proportion of correct prediction of individuals who do not have the health outcome) and sensitivity (proportion of correct predictions of individuals who have the health outcome) of the model were noted. In order to determine possible significant interactions between each predictor and every other predictor, first-order interactions of significant predictors was performed using a backward stepwise entry with a *P* ≤ 0.1 (i.e., all interaction terms are entered into the model and then removed one step at a time). The last step involved entering significant predictors and significant first-order interactions into the model using forward stepwise entry with a *P* ≤ 0.1. This gave rise to a new model that included significant predictors and significant first-order interactions, as well as recalibrated specificity and sensitivity measures. Finally, the model with the highest sensitivity was retained as the best-fit model for each health outcome, thus predicting the highest proportion of individuals who have the health outcome. 

## 3. Results

 Overall, 52 public and 44 private schools participated in the study. Response rate ranged from 9% (private schools in Dubai) to 100% (public schools in Fujairah and private and public schools in western region), resulting in an overall response rate of 65% (Table 1). While undersampling of private schools was relatively high for the Emirate of Dubai and modest for the Emirate of Ajman, the statistically significant relationship between ethnicity (local versus expatriate) and the type of school attended (*χ* ^2^ = 2161.1, *P* = 0.000) implies that stratification by ethnicity is appropriate in meeting the study objectives. The study included 6,329 adolescent student participants with 49% of local national origin. Overall, 44% of the sample was male. The mean age of the sample was 16.2 years. There were significant differences across the emirates in relation to gender with a notably low percentage of males from the UAQ.

### 3.1. Sample Characteristics

Overall, 88% of the students report that their parents are married (Table 2). The majority of expatriate students are Middle Eastern (15.7%), South East Asian (16.8%), and North African Arabs (10%). In relation to socioeconomic status, overall 74.3% of students reported an annual family income below AED15,000, 14.4% reported that their father is unemployed, 57.8% of students reported that their father did not complete high school, 60.5% reported residential crowding, and 51% owned their property of residence. Significant differences across the nine geographic regions were found for all sociodemographic variables except for parental marital status (Table 2).

### 3.2. Residential Profile

Overall, 21.8% of students resided in a property built prior to 1988, 72.2% reported regular use of pesticides or insecticides in their homes, 59.4% reported seeing pests in their residence, 15.6% resided near industry, gas stations, dumpsites, or construction, and 16.1% resided near high power lines or power plants. Significant differences across the nine regions were found for all residential variables with students from the UAQ reporting the highest proportions of residing in older properties and regular exposure to pesticides or insecticides (Table 2). Those from RAK had the highest prevalence of reporting pests in their place of residence and residing in proximity to power lines or power plants. Moreover, 26.4% of students from the western region of Abu Dhabi resided in proximity to industry, gas stations, dumpsites, or construction.

### 3.3. Lifestyle Profile

For each of the nine regions, at least 7.6% reported active smoking, 23.1% reported passive smoking, 0.4% reported ever using illegal drugs, and 22.9% reported using nonconventional substance abuse. The reported mean number of physical activities practiced in the past 12 months was 4.1. Significant differences across the nine regions were found for active and passive smoking, and the number of experienced physical activities (Table 2).

### 3.4. Health Outcomes

Overall, the prevalence of high blood pressure, heart disease, thalassemia, sickle-cell anemia, and iron-deficiency anemia in our sample was 1.8, 1.3, 0.9, 1.6, and 5%, respectively (Table 3). Analysis revealed statistically significant differences among local and expatriate adolescents for diagnosis with thalassemia (*χ* ^2^ = 8.806, *P* = 0.003), sickle-cell anemia (*χ* ^2^ = 15.066, *P* = 0.000), and iron-deficiency anemia (*χ* ^2^ = 29.461, *P* = 0.000). These significant differences were also seen between female locals and female expatriates for thalassemia (*χ* ^2^ = 11.062, *P* = 0.001), sickle-cell anemia (*χ* ^2^ = 5.729, *P* = 0.017), and iron-deficiency anemia (*χ* ^2^ = 18.275,  *P* = 0.000). Among males, significant differences were found between male locals and male expatriates for diagnosis with sickle-cell anemia (*χ* ^2^ = 9.676, *P* = 0.002) and iron-deficiency anemia (*χ* ^2^ = 4.44, *P* = 0.035). On a regional scale, statistically significant differences in the prevalence of iron-deficiency anemia among locals and expatriate student participants were found in Abu Dhabi (*χ* ^2^ = 17.413, *P* = 0.000), western region (*χ* ^2^ = 5.048, *P* = 0.025), and Al-Ain (*χ* ^2^ = 7.377, *P* = 0.007). Finally, significant differences existed between local and expatriate females for diagnosis with iron-deficiency anemia in Abu Dhabi (*χ* ^2^ = 10.176, *P* = 0.001). In relation to diagnosis with high blood pressure, significant differences were found between the local and expatriate population from Sharjah (*χ* ^2^ = 6.965, *P* = 0.008).

### 3.5. Multivariate Analysis

Results of conditional logistic regression modeling for diagnosis with high blood pressure revealed significant predictors of living in proximity to industry, gas stations, dumpsites, or construction; smoking at an earlier age, exposure to smoking at an earlier age, and ever purposely smelling gasoline, glue, fumes, correctors, car exhaust, or burning black ants (Table 4). Results suggested that students from GCC were 11.64 times more likely to develop heart disease, 6.80 times more likely to be diagnosed with sickle-cell anemia, and 3.10 times more likely to be diagnosed with iron-deficiency anemia, when compared with students from south east Asian origin. Local students were more likely to be diagnosed with thalassemia (OR = 7.92), sickle-cell anemia (OR = 4.58), and iron-deficiency anemia (OR = 3.10). Participants who were diagnosed with these blood disorders were also more likely to experience a higher number of physical activities in the past year. Females were more likely to be diagnosed with thalassemia (OR = 3.24) and iron-deficiency anemia (OR = 3.97). 

## 4. Discussion 

Population-based health surveillance data is essential for healthcare and public health planning. This study is the first to examine the prevalence of diagnosis with high blood pressure, heart disease, thalassemia, sickle-cell anemia, and iron-deficiency anemia among the UAE adolescent population. High blood pressure is known to be prevalent in the adult Emirati population aged from 30 to 50 years, with higher prevalence among females than males (53% versus 47%, resp.) [24]. High blood pressure among the adolescent subpopulation is particularly relevant given that it is a leading health risk factor responsible for 2.2–4.9% loss of DALYs, (dependent on a country's income group) and can lead to heart disease [25]. Despite the young age group of the study participants, an overall 1.8% report diagnosis with high blood pressure, and 1.3% report diagnosis with heart disease. In relation to blood disorders, with over 260 known genetic diseases in the UAE, public health efforts have focused on premarital genetic screening in order to decrease the incidence of prevalent blood disorders like sickle-cell diseases, thalassemia, haemophilia, and haemoglobin disorders [26, 27]. Overall, the prevalence of thalassemia, sickle-cell anemia, and iron-deficiency anemia in our sample was 0.9%, 1.6%, and 5%. The latter is largely understudied among UAE adolescents and is in dire need of investigation given the critical role that iron plays in the development of muscle, brain, and red blood cells. An estimated 27% of preschool children worldwide have anemia caused by iron deficiency [28]. Female susceptibility to iron-deficiency anemia is particularly relevant to public health, given that an estimated 18% of maternal mortality in low- and middle-income countries is attributed to iron deficiency [25].

Another important contribution of this study relates to findings concerning potential predictors of these health outcomes. Despite significant differences in demographic, socioeconomic, and lifestyle profiles across the nine regions, geographical region did not emerge as a predictor of the examined health outcomes. Instead, significant predictors of high blood pressure are largely attributed to lifestyle and residential variables such as proximity to industry, gas stations, dumpsites, or construction, age when smoking or exposure to smoking began, and ever purposely smelled gasoline, glue, fumes, correctors, car exhaust, or burning black ants. This is particularly important given that smoking is fairly prevalent in the UAE. Results of the Global Youth Tobacco Survey suggested that 30% of UAE male youths and 13% of females used tobacco at least once or more in the past 30 days [29]. Although, the overall prevalence of active smoking in our sample is 14.7%, it is significantly higher among expatriate males (25%) compared to expatriate females (6%), and it is also linked to ethnicity [30]. These rates are notably high among adolescent expatriates from Arab/Middle Eastern countries (27.7%, 37% among males only) and western origins (27.5%). The same applies for exposure to second-hand smoking with a larger proportion of males exposed to passive smoke (50%) compared to females (31%). 

Significant predictors of heart disease, thalassemia, sickle-cell anemia, and iron-deficiency anemia include ethnicity. In addition, the increased likelihood of participating in a higher number of physical activities among those that report diagnosis with thalassemia, sickle-cell, and iron deficiency anemia can be attributed to these students' quests for an activity that provides both the benefits and comfort. This can be challenging and subject to trial and error given that individuals with anemia tire more easily than others. Significant predictors of iron-deficiency anemia include residing in proximity to industry, age when regular exposure to smoking began, number of physical activities experience in the past year, being local, ethnic background, and sex. Overall, this study suggests that among the adolescent subpopulation, Emirati local adolescents are more likely to be diagnosed with thalassemia, sickle-cell anemia, and iron-deficiency anemia than adolescent expatriates that reside in the UAE. While genetic and ethnic factors may be linked to these differences, other possible explanations may be related to the “healthy migrant effect,” which suggests that immigrants are in general healthier than the host population. Arguments for this phenomenon include health screening by recipient countries, healthy behavior prior to migration, and immigrant self-selection where healthier and wealthier individuals from relatively higher socioeconomic status tend to search for better opportunities in new host countries [31]. Interestingly, variables that did not emerge as significant predictors of high blood pressure, heart disease, thalassemia, sickle-cell anemia, and iron-deficiency anemia include socioeconomic status, parental marital status, father/mother's employment status, residential crowding, father/mother's education level, property tenure, pests in residence, residing near power lines or plants, concern over air pollution, and illegal drug use. 

These results highlight the need for programs and policy efforts that target UAE adolescents and promote awareness related to the role of lifestyle, particularly smoking behavior, and heart health. It is highly likely that the social environment of the UAE promotes smoking behaviour, as evidenced by the increased popularity of shisha or waterpipe in the Arab world, particularly among young male adolescents [32]. Adolescents need to be made aware of the dangers related to smoking and other substance abuse such as purposely smelling gasoline, glue, fumes, correctors, car exhaust, or burning black ants. In addition, knowledge translation in relation to the prevention of iron-deficiency anemia should target the adolescent UAE population, particularly females; results of this study suggest that an appropriate venue would be through the UAE public school system. In relation to thalassemia and sickle-cell anemia, public health efforts in the UAE have led to important developments; for example, in 2006, an independent health centre was established in Dubai to provide professional and quality care for patients with thalassemia, as well as to provide extensive screening, counseling, and prevention policies that aim to reduce the number of newborns affected with thalassemia [27]. Around 40 per cent of the patients at the Dubai Thalassemia Centre are national Emiratis, another 40 per cent are expatriates from Asian and African countries, while the other 20 per cent are Arab nationals [26].

This study was subject to several limitations. First, data are self-reported and may have been subject to response bias. Second, sampling led to lower representations of adolescents that attend private schools in Dubai and among males that reside in the UAQ. This is relevant given that the population of Dubai consists of a large proportion of expatriates; hence, results related to the expatriate population in Dubai are likely to be biased. Despite these limitations, this study contributed to knowledge on the epidemiology of high blood pressure, heart disease, thalassemia, sickle-cell anemia, and iron-deficiency anemia among the UAE adolescent population, as well as knowledge on spatial, gender, and ethnic differences in the diagnosis of these health conditions in the UAE. Future research should continue to examine the health of the adolescent UAE population and the underlying factors linked to their health, in order to facilitate the development of tailored strategies and programs that aim to improve population health.

## 5. Conclusion

Despite the young age group of the study participants, the prevalence of high blood pressure, heart disease, thalassemia, sickle-cell anemia, and iron-deficiency anemia is 1.8, 1.3, 0.9, 1.6, and 5% respectively. Despite significant differences in demographic, socioeconomic, and lifestyle profiles across the nine regions, results suggest that significant predictors of high blood pressure and heart disease are largely attributed to lifestyle and residential variables, while those of thalassemia and sickle-cell anemia include ethnicity and sex. These findings highlight the need for targeted education and awareness programs that focus on the role of lifestyle (particularly smoking behavior), nutrition, and heart health. 

## Figures and Tables

**Table 1:** Distribution and demographic profile of the sample of UAE adolescent participants that reside in the United Arab Emirates (*n* = 6,329).

Sample	Emirate of Abu Dhabi			Dubai	Sharjah	Ajman	RAK	Fujairah	UAQ	Total
	Abu Dhabi	Western	Al-Ain							
Private schools—*n* (%)	11 (69)	5 (100)	6 (86)	2 (9)	10 (71)	2 (40)	3 (50)	3 (60)	2 (67)	**44 (61)**
Public schools—*n* (%)	9 (75)	3 (100)	11 (92)	6 (75)	7 (70)	3 (75)	6 (86)	5 (100)	2 (50)	**52 (80)**
Student participants attending public schools—*n* (%)	756 (52)	192 (51)	956 (75)	432 (73)	516 (44)	180 (68)	459 (68)	394 (76)	111 (76)	**3996 (62)**
Local—*n* (%)										
M	153 (26)	67 (48)	236 (37)	168 (60)	250 (47)	54 (53)	190 (47)	129 (37)	1 (1)	**1248 (40)**
F	442 (74)	72 (52)	398 (63)	112 (40)	285 (53)	47 (47)	216 (53)	218 (63)	87 (99)	**1877 (60)**
Expat—*n* (%)										
M	389 (47)	158 (71)	392 (64)	160 (53)	224 (37)	47 (31)	107 (44)	65 (40)	13 (22)	**1555 (49)**
F	446 (53)	66 (27)	222 (36)	142 (47)	381 (63)	111 (70)	136 (56)	100 (61)	45 (78)	**1649 (51)**
Mean age (SD)—years	16.0 (1.3)	16.1 (1.2)	16.3 (1.2)	16.2 (1.1)	16.1 (1.2)	16.2 (1.2)	16.3 (1.3)	16.1 (1.1)	16.3 (1.2)	**16.2 (1.2)**
Total—*n* (%)	**1430 (23)**	**363 (6)**	**1248 (20)**	**582 (9)**	**1140 (18)**	**259 (4)**	**649 (10)**	**512 (8)**	**146 (2)**	**6329 (100)**
Secondary students in the UAE^†^ (%)	25	<1	6	43	17	4	2	2	<1	100

RAK: Ras al-Khaimah; UAQ: Umm al-Quwain.

^†^Based on student enrolment data from the UAE Ministry of Education, Statistics Department (2005–2007).

**Table 2:** Socioeconomic, demographic, residential, and lifestyle profiles of the student participants (*n* = 6,329).

Variables	AUH	Western	Al-Ain	Dubai	Sharjah	Ajman	RAK	Fujairah	UAQ	Total
Sociodemographic										
Parental marital status (% married)	88.7	92.1	88.2	88.8	87.8	88.7	86.7	85.3	88.0	88.2
Ethnicity**										
UAE local	41.6	38.3	50.4	48.1	46.9	39.0	62.6	67.8	60.3	49.3
GCC	7.8	2.8	4.9	11.5	3.6	12.7	2.3	3.1	6.2	5.8
Arab/Middle East	18.7	24.2	20.5	4.8	15.7	25.5	8.0	10.2	6.8	15.7
Arab/Africa	12.2	12.1	9.7	8.9	9.4	13.1	6.6	9.4	4.1	10.0
South East Asia	16.9	22.6	12.9	25.8	18.2	5.4	19.0	8.6	22.6	16.8
Western	2.0	0	0.4	0	3.6	1.2	0.3	0.6	0	1.3
No nationality	0.1	0	0.1	0	0.9	1.9	0.2	0.2	0	0.3
Others	0.7	0	1.1	0.9	1.7	1.2	1.1	0.2	0	0.9
% family income < AED15,000**	63.5	79.3	77.8	80.7	71.4	90.4	78.1	75.8	80.4	74.3
Father employment**										
Government	47.7	55.1	51.9	42.5	34.8	55.0	48.4	52.7	39.7	46.8
Private	29.4	26.9	17.2	20.3	31.0	20.3	16.3	14.1	18.2	23.2
Self-employed	10.5	8.3	11.5	21.5	20.5	12.6	15.1	7.4	19.8	13.9
Unemployed	10.9	9.7	16.9	14.2	12.5	10.8	18.5	23.8	22.3	14.6
Retired	1.4	0	2.4	1.5	1.1	1.4	1.7	2.0	0	1.5
% father completed high school**	72.1	61.9	53.0	49.1	66.2	55.9	41.1	46.3	35.9	57.8
Residential crowding (%)**	52.4	54.6	59.0	70.0	64.0	69.8	60.1	60.8	76.5	60.5
% own property**	36.1	42.6	52.7	53.8	47.7	36.9	74.2	71.8	70.8	51.0
Residential										
Property built prior to 1988 (%)**	11.1	25.3	22.4	23.1	17.4	15.2	37.8	34.2	39.1	21.8
% regular use of pesticides or insecticides*	70.5	73.3	75.8	73.2	67.9	68.2	72.2	76.3	77.6	72.2
% reporting pests in residence**	53.4	57.7	62.5	60.5	51.2	55.2	70.2	73.2	66.1	59.4
% near industrial, gas stations, dumpsites, and construction**	12.7	26.4	8.0	20.2	15.8	19.2	21.4	14.7	16.5	15.6
% near power lines or plants**	10.8	18.0	18.5	14.8	13.6	19.9	22.6	20.0	18.5	16.1
Lifestyle										
Active smoker (%)**	15.0	19.7	19.3	13.7	15.4	16.7	8.3	12.5	7.6	14.7
Passive smoker (%)**	40.3	43.9	45.8	37.2	40.0	33.3	31.6	38.0	23.1	39.5
Illegal drug use (%)	1.1	1.9	0.4	1.9	2.2	3.6	2.2	1.9	1.5	1.6
Nonconventional substance abuse (%)	31.8	22.9	25.4	27.6	34.7	27.2	24.9	29.0	42.1	29.4
Physical activity (mean-types practiced)^∗∗∧^	4.2	4.6	4.1	3.9	4.2	3.8	4.0	4.0	3.2	4.1

*P < 0.01; **P < 0.001.

^∧^(*F*_1,8_ = 3.971, *P* < 0.0001).

**Table 3:** Prevalence of medical diagnoses among the UAE adolescent participants (*n* = 6,329).

	Medical condition (%)																																		
Region	High blood pressure/hypertension							Heart disease							Thalassemia							Sickle-cell anemia							Iron-deficiency anemia						
	Local			Expatriate			T	Local			Expatriate			T	Local			Expatriate			T	Local			Expatriate			T	Local			Expatriate			T
	M	F	T	M	F	T		M	F	T	M	F	T		M	F	T	M	F	T		M	F	T	M	F	T		M	F	T	M	F	T	
AUH	2.0	2.0	2.0	1.9	2.7	2.3	2.2	2.0	1.4	1.5	1.1	1.1	1.1	1.2	0.7	2.3	1.8	0.8	0.2	0.5	1.1	3.9	1.6	2.2	0.8	2.7	1.8	2.0	2.0	12.4	9.9***	1.9	6.4	4.3***	6.6
WES	4.2	0	1.7	4.4	4.0	4.3	3.3	2.1	4.2	3.4	1.5	2.0	1.6	2.3	0	1.4	0.8	0	2.0	0.5	0.7	0	4.2	2.5	0.7	2.0	1.1	1.7	2.1	12.7	8.5*	2.2	4.1	5.0*	5.0
Al-Ain	1.3	0.9	10	1.1	0.9	1.1	1.1	0.9	1.2	1.0	1.0	0.0	0.7	1.0	0.4	1.2	0.9	0.6	1.0	0.7	0.8	0.9	2.6	1.9	0.6	0.5	0.5	1.2	1.7	10.1	6.4**	1.3	6.3	3.0**	5.1
Dubai	3.3	2.7	3.0	2.7	1.4	2.1	2.5	0.7	1.8	1.1	2.7	0.7	1.7	1.4	1.3	0.9	1.1	0.7	0.0	0.3	0.7	3.9	1.8	3.0	0.0	0.0	0.0	1.4	5.3	8.9	6.8	2.7	4.2	3.4	5.1
Sharjah	3.7	2.8	3.2**	0.4	1.3	1.0**	2.0	2.4	1.8	2.1	1.5	1.6	1.5	1.8	2.0	1.1	1.5	0.9	0.3	0.5	1.0	1.2	3.9	2.6	0.4	0.3	0.3	1.4	2.4	6.7	4.9	1.3	3.9	3.0	3.8
Ajman	1.9	0.0	1.0	0.0	0.0	0.0	0.4	1.9	2.1	2.0	0.0	0.0	0.0	0.8	1.9	6.4	4.0	0.0	0.9	0.6	0.7	7.5	4.3	6.0	4.3	0.9	1.9	3.5	3.8	14.9	9.0	0.0	3.6	2.5	5.0
RAK	2.1	0.5	1.2	0.0	0.7	0.4	0.9	1.6	0.9	1.2	0.9	0.0	0.4	0.9	0.0	0.9	0.5	1.9	0.0	0.8	0.6	1.1	2.3	1.6	1.9	0.7	1.2	1.5	2.6	6.5	4.7	1.9	4.4	3.3	4.2
FUJ	1.6	0.9	1.2	3.1	2.0	2.4	1.6	0.8	0.9	0.9	3.1	1.0	1.8	1.2	0.0	1.4	0.9	0.0	0.0	0.0	0.6	2.3	0.9	1.4	0.0	2.0	1.2	1.4	3.1	5.0	4.3	1.5	4.0	3.0	3.9
UAQ	0.0	0.0	0.0	7.7	4.4	2.1	2.1	0.0	0.0	0.0	0.0	0.0	0.0	0.0	0.0	0.0	0.0	0.0	0.0	0.0	0.0	0.0	0.0	0.0	0.0	0.0	0.0	0.0	0.0	2.3	2.3	0.0	8.9	6.9	4.1

Total	2.4	1.4	1.8	1.7	1.7	1.7	1.8	1.5	1.4	1.4	1.3	0.9	1.1	1.3	0.8	1.5**	1.2**	0.7	0.4	0.5**	0.9**	2.2**	2.3*	2.2***	0.7**	1.2*	1.0***	1.6	2.7	8.9	6.5**	1.7	5.1	3.4**	5.0

*P < 0.05; **P < 0.01; ***P < 0.001.

T: total sample; AUH: Abu Dhabi; WES: western; RAK: Ras al-Khaimah; FUJ: Fujairah; UAQ: Umm al-Quwain.

**Table 4:** Predictors of high blood pressure, heart disease, thalassemia, sickle-cell anemia, and iron-deficiency anemia among the UAE adolescent population.

Variables	Reference	High blood pressure		Heart disease		Thalassemia		Sickle-cell anemia		Iron-deficiency anemia	
		OR (95% CI)	*P*	OR (95% CI)	*P*	OR (95% CI)	*P*	OR (95% CI)	*P*	OR (95% CI)	*P*
Proximity to industry (no)	Yes	**2.86 (1.63–5.03)**	0.000	1.96 (0.85–4.56)	0.115	1.27 (0.47–3.49)	0.638	0.64 (0.25–1.63)	0.344	**1.69 (1.12–2.55)**	0.012
Nonconventional drugs (no)	Yes	**2.43 (1.39–4.25)**	0.002	1.68 (0.77–3.68)	0.194	1.49 (0.65–3.46)	0.349	1.28 (0.68–2.44)	0.447	1.17 (0.82–1.68)	0.387
Age when smoking began	Increasing	**0.99 (0.98–0.99)**	0.027	0.99 (0.98–1.01)	0.652	1.00 (0.98–1.02)	0.964	1.00 (0.99–1.01)	0.988	1.00 (0.99–1.01)	0.521
Age when regular exposure to smoking began	Increasing	**0.99 (0.98–0.99)**	0.008	0.99 (0.98–1.00)	0.162	1.00 (0.99–1.01)	0.972	1.00 (0.99–1.01)	0.280	**0.99 (0.99–0.99)**	0.006
Physical activities practiced (number in past 12 months)	Increasing	1.06 (097–1.15)	1.060	1.08 (0.97–1.21)	0.174	**1.20 (1.08–1.34)**	0.001	**1.12 (1.03–1.22)**	0.009	**1.06 (1.00–1.13)**	0.036
Ethnic (expat)	Local	0.768 (0.367–1.606)	0.483	3.49 (0.79–15.46)	0.099	**7.92 (1.05–60.01)**	0.045	**4.58 (1.38–15.20)**	0.013	**3.10 (1.71–5.65)**	0.000
Ethnic (SE Asian)			0.812		0.002		0.591		0.004		0.012
Other GCC		0.48 (0.10–2.25)	0.352	**11.64 (2.26–59.93)**	0.003	—	0.996	**6.80 (1.59–29.13)**	0.010	**3.26 (1.35–7.86)**	0.009
Arab/Middle Eastern		0.79 (0.34–1.87)	0.600	1.87 (0.33–10.72)	0.482	4.73 (0.51–43.95)	0.172	0.43 (0.04–4.21)	0.469	1.97 (0.96–4.06)	0.065
North African		0.79 (0.30–2.11)	0.640	0.84 (0.07–9.39)	0.885	4.12 (0.37–46.12)	0.251	**6.16 (1.64–23.18)**	0.007	**3.05 (1.48–6.28)**	0.002
Sex (male)	Female	1.19 (0.65–2.17)	0.573	1.83 (0.76–4.14)	0.178	**3.24 (1.16–9.07)**	0.025	1.35 (0.69–2.65)	0.377	**3.97 (2.54–6.20)**	0.000
